# Latest Advancements in the Development of High-Performance Lignin- and Tannin-Based Non-Isocyanate Polyurethane Adhesive for Wood Composites

**DOI:** 10.3390/polym15193864

**Published:** 2023-09-23

**Authors:** Apri Heri Iswanto, Muhammad Adly Rahandi Lubis, Jajang Sutiawan, Syeed Saifulazry Osman Al-Edrus, Seng Hua Lee, Petar Antov, Lubos Kristak, Roman Reh, Efri Mardawati, Adi Santoso, Sukma Surya Kusumah

**Affiliations:** 1 Department of Forest Product, Faculty of Forestry, Universitas Sumatera Utara, Medan 20155, Indonesia; jajangsutiawan@usu.ac.id; 2 Research Center for Biomass and Bioproducts, National Research and Innovation Agency, Cibinong 16911, Indonesia; marl@biomaterial.lipi.go.id (M.A.R.L.); adis001@brin.go.id (A.S.); sukma.surya@biomaterial.lipi.go.id (S.S.K.); 3 Research Collaboration Center for Biomass and Biorefinery between BRIN and Universitas Padjadjaran, National Research and Innovation Agency, Bandung 40600, Indonesia; efri.mardawati@unpad.ac.id; 4 Institute of Tropical Forestry and Forest Products, Universiti Putra Malaysia (UPM), Serdang 43400, Malaysia; saifulazry@upm.edu.my; 5 Department of Wood Industry, Faculty of Applied Sciences, Universiti Teknologi MARA (UiTM), Kampus Jengka, Pahang 26400, Malaysia; leesenghua87@gmail.com; 6 Faculty of Forest Industry, University of Forestry, 1797 Sofia, Bulgaria; p.antov@ltu.bg; 7 Faculty of Wood Sciences and Technology, Technical University in Zvolen, 96001 Zvolen, Slovakia; kristak@tuzvo.sk (L.K.); reh@tuzvo.sk (R.R.); 8 Department of Agro-Industrial Technology, Universitas Padjadjaran, Jatinangor 40600, Indonesia

**Keywords:** bio-based polyol, adhesives, non-isocyanate, renewable, thermosetting resin

## Abstract

The depletion of natural resources and increasing environmental apprehension regarding the reduction of harmful isocyanates employed in manufacturing polyurethanes (PUs) have generated significant attention from both industrial and academic sectors. This attention is focused on advancing bio-based non-isocyanate polyurethane (NIPU) resins as viable and sustainable substitutes, possessing satisfactory properties. This review presents a comprehensive analysis of the progress made in developing bio-based NIPU polymers for wood adhesive applications. The main aim of this paper is to conduct a comprehensive analysis of the latest advancements in the production of high-performance bio-based NIPU resins derived from lignin and tannin for wood composites. A comprehensive evaluation was conducted on scholarly publications retrieved from the Scopus database, encompassing the period from January 2010 to April 2023. In NIPU adhesive manufacturing, the exploration of substitute materials for isocyanates is imperative, due to their inherent toxicity, high cost, and limited availability. The process of demethylation and carbonation of lignin and tannin has the potential to produce polyphenolic compounds that possess hydroxyl and carbonyl functional groups. Bio-based NIPUs can be synthesized through the reaction involving diamine molecules. Previous studies have provided evidence indicating that NIPUs derived from lignin and tannin exhibit enhanced mechanical properties, decreased curing temperatures and shortened pressing durations, and are devoid of isocyanates. The characterization of NIPU adhesives based on lignin and tannin was conducted using various analytical techniques, including Fourier-transform infrared spectroscopy (FTIR), thermogravimetric analysis (TGA), matrix-assisted laser desorption/ionization with time-of-flight (MALDI-TOF) mass spectrometry, and gel permeation chromatography (GPC). The adhesive performance of tannin-based NIPU resins was shown to be superior to that of lignin-based NIPUs. This paper elucidates the potential of lignin and tannin as alternate sources for polyols in the manufacturing of NIPUs, specifically for their application as wood adhesives.

## 1. Introduction

Since 2014, the global market for wood-based panels has consistently expanded, with production exceeding 370 million m^3^ by 2021 [1,2]. Phenol-formaldehyde (PF), urea-formaldehyde (UF), and melamine-urea-formaldehyde (MUF) are the main synthetic adhesives used in the production of wood-based panels [3,4,5]. These adhesives are extensively used due to their diverse properties, such as flexibility, low cost, and excellent thermal stability [6]. However, formaldehyde emissions have piqued the interest of those seeking ecologically sustainable and safe alternatives to wood adhesives [7]. In light of the formaldehyde restrictions and the rising environmental consciousness, the adhesive industry must become more sustainable and environmentally friendly, and less reliant on fossil fuels. According to the material safety data sheet (MSDS), formaldehyde is a carcinogenic and toxic substance with an acute oral toxicity (LD50) of 100 mg/kg (Rat) [8]. According to Demilec LLC’s MSDS, pMDI is significantly less toxic than formaldehyde, with an acute oral toxicity (LD50) of >2000 mg/kg (Rat) [8].

Consequently, developing formaldehyde-free bio-adhesives derived from waste streams and non-usable materials is viewed as an innovative solution with a significant market presence [2,9]. However, bio-based adhesive formulation for the wood-based panel industry is still at the laboratory scale [2], so scaling up to a larger production capacity is required to evaluate and compare the market potential of bio-adhesives [2,10]. To successfully replace fossil-based adhesives with sustainable alternatives, the adhesive characteristics and bonding performance must be extremely similar, or offer added value, and the cost performance must be comparable to existing non-sustainable adhesives [11].

Bayer and his colleagues discovered polyurethanes (PUs) in 1937, by combining a polyester diol with a diisocyanate, making diisocyanate one of the most frequently manufactured substances in the world [12]. The global market for isocyanates is expanding at a rate of 5% per year, primarily due to increased polyurethane production. Toluene diisocyanate (TDI) and methylene diphenyl isocyanate (MDI), which are used to produce flexible and rigid polyurethane products, respectively, are simple precursors that enable the production of a diverse range of products [13]. Thermoplastic elastomers and thermoset resins (including foams) are marketed daily as essential components in a variety of products, such as paints, binders, and aerospace materials, dependent on whether they are created using linear or cross-linked networks. Even in viscous conditions or at low temperatures, isocyanate is advantageous due to its remarkable reactivity and high reaction yield.

The vast range of mechanical properties that these materials can exhibit is primarily the result of two physical–chemical processes: phase separation between hard and flexible segments and hydrogen bonding between urethane linkages [12,13,14,15]. Due to its remarkable reactivity and high reaction yield, isocyanate chemistry is advantageous even at low temperatures and in viscous conditions. It has been reported that common isocyanates such as MDI and TDI are dangerous because they can infiltrate the body through inhalation, skin (open wounds), eye contact, or the mouth, by ingesting contaminated food or drink, or by smoking [16,17,18]. In addition, the reaction of phosgene with amines is utilized in the industrial production of isocyanate. The gas phosgene is odorless, intensely reactive, and extremely toxic. This hazardous gas can cause severe respiratory symptoms, as well as ocular irritation, burns to the eyes and skin, and even mortality. 

In addition, there is a demand for environmentally friendly operations and products due to the need to safeguard the welfare of both employees and consumers. These issues have prompted the development of alternative polyurethane synthesis techniques. In the preponderance of alternative polyurethane production processes, carbon dioxide is utilized directly or indirectly as a sustainable feedstock. Non-isocyanate polyurethanes (NIPUs) can be manufactured using one of three primary techniques: step-growth polyaddition, polycondensation, or ring-opening polymerization. Recent years have seen a surge in commercial and academic interest in the development of NIPUs for renewable resources [19,20]. NIPUs can be produced from polyols derived from renewable resources, including lignin [3,21,22,23,24,25,26,27,28] and tannin [3,24,29,30,31,32,33,34,35,36,37]. Because lignin and tannin are natural polymer molecules derived from plants, they are the most prevalent renewable adhesive sources. Another issue with lignin and tannin is the frequently large property variation resulting from extraction and fractionation techniques, which impacts the quality and cost of adhesives.

**Figure 1:**
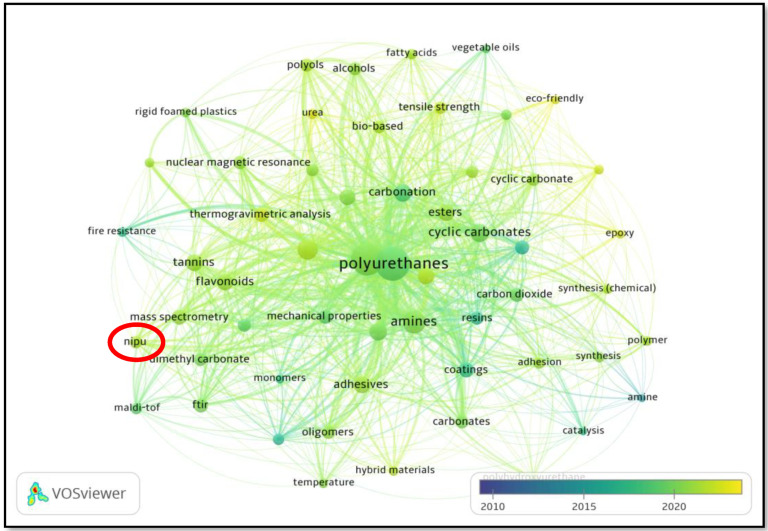
Overlay visualization on the progress of research into bio-based NIPU resins during 2010–2023 using VOSviewer (version 1.6.18). The red circle indicates that NIPU has received less attention in recent years compared to conventional PU resins.

This review article examines scholarly articles published between January 2010 and April 2023 in the Scopus database on the topic of bio-based NIPU adhesives derived from lignin and tannin from various perspectives and methodologies (Figure 1). Using Mendeley Desktop (version 1.19.8, Mendeley Ltd., New York, NY, USA), all duplicates and non-English documents were eliminated. The systematic review was conducted so that the materials’ relevance could be determined. Polyurethane, non-isocyanate polyurethane, cyclic carbonates, polyhydroxy urethane, flavonoids, dimethyl carbonates, and others were identified as keyword variations. The grouping of keywords corresponds to the primary objective of this review, which is based on a thorough examination of the existing literature in order to select the types of bio-based NIPU adhesives on which the research has focused. Consequently, non-isocyanate polyurethane was selected as the standard term for these published keywords. After grouping the diverse keywords, the diversity of terms used to refer to the same article has been considerably reduced, resulting in a more appropriate and clearer graphical representation. The center point in the graph indicates that the primary research keyword is PU. The study conducted near to the center of PU focuses on the advancement of PU adhesives. This includes the cyclic carbonates, amines, and carbonation, as well as the examination of the mechanical properties exhibited by PU. In contrast, the positioning of the phrase NIPU is situated at a considerable distance from the central area, suggesting a scarcity of research endeavors undertaken within the preceding decade.

Overall, it is challenging to replace the conventional PU adhesive with well-known and repeatable properties with bio-based polymers that have a wider range of industrial applications [3,33,38,39,40,41]. The development of NIPU adhesives faces additional obstacles; adhesives made from bio-based polymers frequently exhibit poor water resistance and low reactivity, and are too expensive to compete with conventional PU adhesive. As a consequence, the primary objective of this review paper is to provide a systematic overview of the most recent advances in the development of high-performance bio-based NIPU adhesives derived from lignin and tannin for wood-based composites.

## 2. Preparation of NIPU Resins for Wood Adhesive

**Figure 2:**
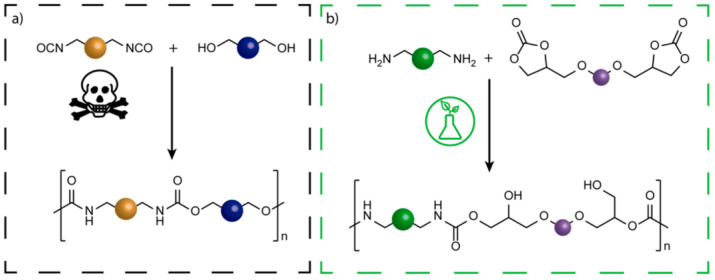
Comparison of conventional step-growth polymerization of PU (**a**) and polyaddition of bis (cyclic carbonate) and diamine to produce NIPU (**b**) [46]. Open-access CC-BY 4.0.

It is necessary to develop alternative PU synthesis technologies that are both environmentally safe and safe for humans. Substituting fossil fuel-derived feedstocks with sustainable bio-based resources is one of the alternative green routes for PU synthesis, which is associated with a reduction in petrochemical processes and the use of fossil fuels (Figure 2). Bio-polyols and bio-based chain extenders have been investigated and developed for use in the synthesis of PU [7,39,42]. DuPont, BASF, BioAmber, Myriant, General Mills Co., Covestro, Henkel Corporation, and Allessa are among the most prominent producers of bio-based raw materials for PU synthesis [3]. Aside from this, considerable efforts have been made to develop a phosgene-free and isocyanate-free synthesis method [3,12,22,43,44,45]. Non-isocyanate polyurethane (NIPU) synthetic methods eschew the use of toxic and moisture-sensitive diisocyanates, which are produced by phosgene. According to the published literature, four distinct synthetic methods for NIPU resin synthesis have been identified, namely step-growth polyaddition, polycondensation, ring opening polymerization (ROP), and rearrangement processes [3,9,12,39,44,45,46,47]. The rearrangement mechanisms promote adhesive curing at low temperatures, stronger adhesive bonds, high thermal stability, and wet and shear resistance [2].

The viability of utilizing Hoffman, Curtius, and Lossen rearrangements was proved in the published research articles [39,46,48]. Regrettably, the use of substrates such as carboxamides, acyl azides, and hydroxamic azides is limited in the synthesis of non-isocyanate polyurethanes due to their inherent toxicity. The reactants employed in the process of ring-opening polymerization are likewise a subject of apprehension. Aliphatic cyclic carbamates are typically synthesized from very hazardous compounds such as phosgene and aziridines [39,46,48]. The polycondensation pathway is characterized by the chemical reactions that occur between di- or polycarbamates and diols, di- or polycarbonates and amino alcohols or diamines, polychloroformate and polyamine, and polycabramoyl chloride and polyol [3,12,44,45]. The limited commercial relevance of this procedure is attributed to the generation of byproducts such as H_2_O, HCl, and alcohols. In addition, syntheses are frequently conducted in solvents, utilizing catalysts, and are subject to certain reaction conditions, such as prolonged reaction durations or elevated temperatures (ranging from 150 to 220 °C), which are deemed economically unfavorable in an industrial context. 

The synthetic technique for the manufacture of NIPUs that has been extensively explored and is considered the most optimal involves the polyaddition of bifunctional cyclic carbonates with primary di- or polyamines (Figure 2b). Cyclic carbonates are commonly regarded as the principal components utilized in polyaddition reactions with diamines. There exist three primary synthetic pathways for the production of these reactants: (i) the process of transesterification involving diols and alkylene carbonates, (ii) the carboxylation of epoxy precursors using CO_2_ in the presence of a catalytic system, and (iii) the synthesis of cyclic carbonates from diols through reaction with CO_2_ in the presence of a catalytic system [14]. The polyaddition of cyclic carbonates and polyamines results in the formation of poly(hydroxy urethane)s (PHUs) that possess extra hydroxyl groups, either primary or secondary, in the side chain [49,50,51,52]. The hydroxyl groups that are produced have the ability to establish hydrogen bonds with both intermolecular and intramolecular urethane carbonyl groups. An additional benefit of the polyaddition method is the utilization of environmentally sustainable bio-based resources and the fixation of carbon dioxide. Moreover, syntheses can be conducted without the requirement of catalysts or solvents [53,54,55,56]. Consequently, the resulting polymeric hybrid materials exhibit enhanced chemical resistance against organic solvents. In addition, it should be noted that polymeric hydrazine urea compounds have enhanced thermal stability due to the absence of labile biuret and allophanate groups, which are known to be thermally unstable. One additional benefit of primary hydroxyl groups is their ability to engage in chemical reactions with diverse functional groups. However, as summarized in Table 1, conflicting findings are found on the water absorption of NIPU in comparison to normal PU [12,38,43,46,57]. 

It is noteworthy that Nanotech Industries, Inc. holds the distinction of being the pioneering entity for effectively implementing NIPUs [3,56,57,58]. The company holds a patent for Green PolyurethaneTM, a commercially available product that is free from phosgene and isocyanate [56]. The bio-resins are produced through the utilization of bio-based materials by means of the reaction between cyclic carbonates, aliphatic polyamines and cycloaliphatic polyamines. Bio-sourced polyamines can be synthesized from amino acids, such as arginine, ornithine, putrescine, spermidine, spermine, and cadaverine [59]. The aforementioned materials exhibit comparable adhesion, hydrolytic stability, and resistance to chemical degradation, corrosion, and wear, in comparison to the conventional commercially available PU adhesive [57]. The progressive exhaustion of fossil resources could potentially lead to a limited availability of materials for the production of polymers. The application of bio-based, renewable monomers, such as vegetable oils, fatty acids, sugar, terpenes, and their derivatives, as well as the conversion of carbon dioxide, has seen ongoing and expanding usage in recent years [60]. 

### 2.1. Lignin-Based NIPU Adhesives

The use of kraft lignin (KL) as a polyol alternative within the NIPU matrix has the potential to enhance the aromatic composition of the resultant polymer. KL exhibits a degree of stability at elevated temperatures and functions as a thermosetting material, owing to its aromatic and crosslinked nature [10,23,25,28,61,62]. Consequently, it is anticipated that the incorporation of lignin will enhance the glass transition temperature and thermal stability of the adhesive. The presence of sulfur in lignin, specifically those that are organically linked, such as thiol (–SH), sulfide bonds (–S–), or disulfide bonds (–S–S–) can reduce the lignin’s thermal stability [63]. Furthermore, the presence of sulfur-containing compounds in the lignin-based NIPU has facilitated the generation of volatile sulfur compounds during thermal degradation [21]. The content, chain length, and number of hard and soft segments have a significant impact on the glass transition temperature, thermal degradation, and mechanical strength of lignin-based NIPUs [13,15,61,62]. The shear strength exhibited a decrease with an increase in the chain length of polyethylene glycol (PEG), with the most favorable outcomes observed for NIPUs containing 50% *w*/*v* lignin in polyol (specifically, PEG with a molecular weight of 200 g/mol) [64]. In addition, it was observed that with an increase in lignin concentration to 50% *w*/*v* of polyol, the first decomposition temperature exhibited an increase from 270 to 285 °C [13,15,61,62]. The mechanical properties of PU are predominantly governed by the degree of crosslinking, which is dictated by the stoichiometry and functionality of the reactants involved. The inclusion of lignin in the NIPU structure, specifically in the soft part, results in its substitution for polyol. Lignin can hydrogen bond with C–O and N–H inside the hard segment, and with C−O−C within the soft segment of PU [65]. Consequently, the glass transition temperature of the lignin-based NIPU exhibited a greater thermal stability compared to that of the conventional PU. However, due to its aromatic properties, lignin primarily serves as the hard segment, hence enhancing the mechanical strength of the NIPU [21]. 

**Figure 3:**
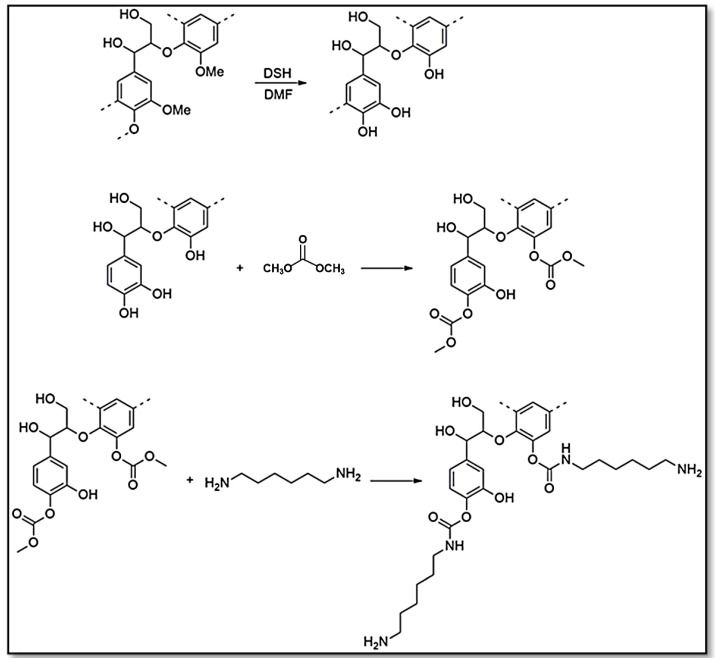
Possible reaction pathway of lignin-based NIPUs through demethylation using 1-dodecanethiol (DSH) and dimethylformamide (DMF) and carbonation using dimethyl carbonate (DMC) [3]. Open-access CC-BY 4.0.

The presence of an aromatic structure in lignin contributes to the enhancement of mechanical strength and thermal properties in blends, copolymers, and composite materials [21]. The ultimate properties of the adhesive will be influenced by the quantity and chemical composition of the lignin employed [22]. Recent studies have indicated that the use of lignin in the polyurethane matrix has the potential to greatly enhance thermal stability, delamination resistance, and abrasion resistance [22,23,24,28]. Nevertheless, research has demonstrated that exceeding a lignin content of 60% can lead to increased brittleness and the occurrence of phase separation [66]. Separation between the soft and hard segments typically causes the glass transition of the soft segment to occur at a low temperature. Furthermore, this negatively affects stress transmission and ultimately compromises the mechanical properties of the lignin-based NIPU [10,61,67]. One of the primary obstacles hindering the wider use of lignin and lignin-derived compounds in condensation adhesives is their limited reactivity towards condensation processes [28,32,68]. Several ways of modifying lignin have been demonstrated to enhance its reactivity. These strategies include demethylation [3,22,69], hydroxy-methylation [3,22], phenolation [3,21,22,70,71], and oxidation [3,9,21,22,52,62,72,73]. Demethylation emerges as a viable and encouraging technology within this context, since it facilitates the conversion of certain aromatic methoxy groups into phenolic hydroxyl groups under conditions of mild reactivity (Figure 3).

Based on several studies, the traditional papermaking technique is frequently utilized in the treatment of raw lignin during the demethylation process. This process involves a nucleophile substitution reaction between hydroxyl and sulfite ions, resulting in the conversion of lignin into sulfite pulp [3,9,21]. In addition, sulfite ions are capable of undergoing nucleophilic substitution events that lead to the cleavage of aryl methyl ethers present in aromatic compounds. This process causes a decrease in the concentration of methoxy groups and enhances the reactivity of the modified lignin. Figure 3 illustrates a potential mechanism for the conversion of lignin into NIPUs. The potential application of the combination of 1-dodecanethiol (DSH) and dimethylformamide (DMF) for the demethylation of lignin is being considered. The proposal suggests that demethylated lignin, due to its higher concentration of phenolic hydroxyl groups, holds potential for the construction of NIPUs. The utilization of dimethyl carbonate (DMC) in carbonation can yield carbonyl groups that serve as potential precursors for the synthesis of NIPUs. The subsequent creation of NIPUs involves the incorporation of hexamethylene diamine (HMDA) [3]. Demethylation of lignin may increase the selectivity and reactivity of the subsequent reaction with DMC. By removing the methyl groups, steric hindrance is reduced and the accessibility of hydroxyl groups for carbonation with DMC is increased. The carbonated lignin then reacts with the diamide to form urethane linkages of NIPU. This can lead to a more controlled and efficient reaction [21].

The demethylation process has the potential to enhance the reactivity of lignin by changing certain methoxy groups into phenolic hydroxyl groups by a nucleophilic substitution reaction. Consequently, this transformation leads to the creation of additional reactive sites on the lignin structure. In order to enhance the adhesive properties of demethylated lignin, the reaction system was supplemented with sodium periodate [74]. The presence of demethylated lignin is responsible for its distinct characteristics. Consequently, the outcome is a more interconnected and reinforced network, exhibiting enhanced adhesive properties and increased resistance to water. The maximal adhesion strengths of glued wood specimens are achieved when the concentration of sodium periodate reaches 20%. This adhesive exhibits a substantial biomass content, is relatively straightforward to synthesize, cost-effective, and well-suited for bonding wood [21,22,61,74]. The reduction in the concentration of non-condensable organic compounds in the final wood adhesive resulted in a corresponding decrease in its toxicity. In contrast to conventional PU, the NIPU exhibited a reduced setting time and enhanced adhesion to the wooden substrate, leading to a prolonged setting time but elevated shear strength measurements [21,22,61]. The aromatic rings inside the lignin structure contribute to the enhancement of film toughness. It can be observed that the thermal properties of NIPU adhesive derived from lignin exhibit a positive correlation between lignin concentration and both the molecular weight and glass transition temperature of the system. The increase can be attributed to the cross-linking effect of lignin-based NIPU with HDMA (Figure 3).

### 2.2. Tannin-Based NIPU Adhesives

**Figure 4:**
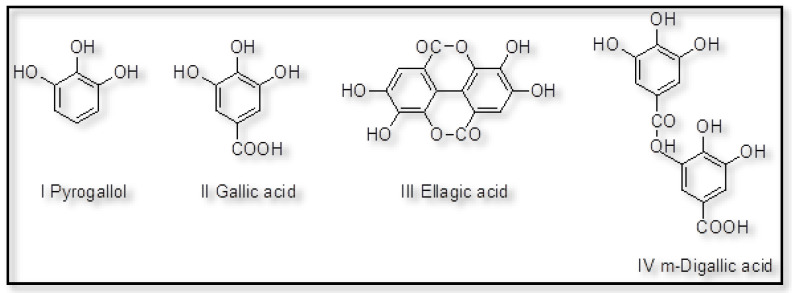
Simple phenol structures in tannin [76]. Open-access CC-BY 4.0.

Tannins can be classified into two categories: hydrolysable tannins, which include gallotannins and ellagitannins, and condensed molecules known as polyflavonoid tannins, with global tannin production reaching 1.1 million tonnes in 2017 [75]. Hydrolysable tannins refer to derivatives of gallic acid that are classified according to the outcomes of their hydrolysis, as depicted in Figure 4 [76]. Consequently, these compounds can be categorized as gallotannins, which predominantly comprise gallic acid and glucose, and ellagitannins, which largely consist of digallic and ellagic acids, as well as penta-galloyl-glucose. The constituents of these entities encompass a diverse array of combinations, with the principal constituents comprising oligomers of n-galloyl-glucose and its rearrangement derivatives, namely vescalin and vescalagin. The primary recurring constituent within hydrolysable tannins is pentagalloyl glucose, which is a polyester synthesized from gallic acid and hexahydroxy diphenic acid. The condensed tannin extracts consist of flavonoid units that have undergone varying degrees of condensation [30,31,33]. The quantities of monoflavonoids and nitrogen-containing acids present in the extract are insufficient to exert any discernible influence on its overall chemical and physical characteristics. Adequate quantities of simple carbohydrates, such as hexoses, pentoses, and disaccharides, as well as complex glucuronates in the form of hydrocolloid gums, are commonly found. Additionally, oligomers resulting from the hydrolysis of hemicelluloses are also often present. In a similar manner, tannins occasionally exhibit the presence of carbohydrate chains with diverse lengths that are linked to flavonoid units. Furthermore, it is commonly acknowledged that tannins can undergo various modifications, such as hydrolysis, polymerization, copolymerization, or acetylation, resulting in the creation of novel materials [3,24,30,31,32,33,35,36,37]. The carbonated derivatives of gallic acid and tannic acid were synthesized using the carbonation process with DMC [3,12]. The cyclic carbonates that were acquired were further subjected to a curing process using amines, resulting in the creation of novel bio-based NIPUs derived from natural polyphenolic waste materials.

**Figure 5:**
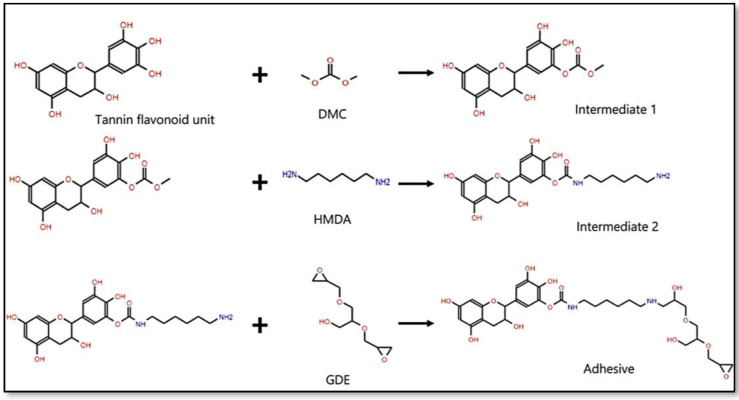
Schematic representation of the reaction of preparation of tannin-based NIPU obtained by reaction of hydroxyl groups of tannin units with DMC followed by reaction with HMDA and glycerol diglycidyl ether (GDE) [2,76]. Open-access CC-BY 4.0.

The development of NIPU resins has been undertaken in order to eliminate the presence of toxic isocyanates during their preparation. This is achieved through a two-step process involving the reaction of synthetic polyols with cyclic or bicyclic carbonates, followed by the reaction with diamines. It is worth noting that some of diamines represent the seminal approach and pioneering work in these significant techniques [31,43,44,77,78]. NIPUs have been created by employing a diverse range of bio-based components in addition to synthetic elements. In recent times, tannins have been utilized in the production of NIPUs for a range of purposes, with a particular focus on surface coatings. The reaction to polyhydroxy urethanes is predicated upon the interplay between condensed tannin and dimethyl carbonate (or more intricate cyclic carbonates), afterwards succeeded by the reaction of the carbonated tannin with a diamine molecule, as depicted in Figure 5. The investigation revealed that the formation of NIPUs was not solely attributed to tannins, but also involved the interaction of carbohydrates present in tannin extracts. This phenomenon was particularly observed in hydrolysable tannins, which possess a carbohydrate-rich backbone. The researchers were motivated by this finding to endeavor synthesizing NIPUs from glucose and sucrose in order to explore their potential applications [35,70,73,79,80]. Therefore, bio-based NIPUs were synthesized predominantly using glucose and sucrose as starting materials by reactions involving DMC and HMDA. Subsequently, the oligomers of the resultant NIPUs were characterized. The glucose NIPU resins were assessed for their suitability as thermosetting adhesives for wood joints, while the sucrose NIPUs were examined for their potential as adhesives for particleboards [77,78].

Tannin-derived NIPUs were synthesized through the reaction between condensed flavonoid tannins and DMC. Subsequently, the combinations underwent treatment with HMDA in order to form urethane linkages. The analysis of the resulting materials involved the utilization of FTIR spectroscopy [78,79,80,81,82,83,84,85,86,87,88], MALDI-TOF mass spectrometry [22,77,79], and TGA [78,79]. The findings from these techniques confirmed that the obtained materials are polyurethanes. Based on the findings of the FTIR analysis, it is unequivocally shown that the chemical compounds generated through the application of heat to tannins are indeed polyurethanes. The adhesives composed of tannin-based NIPUs exhibit insolubility, indicating that they undergo curing and form three-dimensional polymeric cross-linkages within a portion of their overall structure [89,90]. Nevertheless, it is possible to dissolve and subject a fraction of their mass, which consists of oligomers polyurethane, to analysis using MALDI-TOF spectrometry. This analytical technique enables the identification and confirmation of the existence of carbonated and/or urethane functional groups within the sample. The TGA results indicated that the degradation of the PU occurs within the temperature range of 180 to 200 °C [78,79].

## 3. Characterization of Bio-Based NIPU Adhesive for Wood-Based Composites

The characterization of bio-based NIPU resins encompasses a variety of tests. Multiple analytical techniques were employed to analyze bio-based NIPU resins, including Fourier-transform infrared spectroscopy (FTIR), thermogravimetric analysis (TGA), matrix-assisted laser desorption/ionization time-of-flight mass spectrometry (MAL-DI-TOF), carbon-13 nuclear magnetic resonance spectroscopy (^13^C-NMR), and gel permeation chromatography (GPC). Fourier-transform infrared spectroscopy (FTIR) is a fundamental analytical tool used to examine the functional groups present in bio-based NIPU, as indicated in Table 2. The peaks seen at wavelengths of 3330 cm^−1^ and 1540 cm^−1^ correspond to N-H stretching vibrations and deformation vibrations of lignin- and tannin-based NIPUs, respectively. Additionally, a notable absorption peak is observed at 1720 cm^−1^, which can be attributed to the stretching vibration of the C=O functional group. The spectra of tannin-based NIPUs display a significant absorption band ranging from 3500 cm^−1^ to 3100 cm^−1^, which can be attributed to the stretching vibration of –OH [35]. The stretching of the N-H group linked to urethane, resulting from the reaction between tannins, DMC, and HMDA, was identified and characterized by the absorption band observed at 3337 cm^−1^. Moreover, two absorption bands associated with the stretching vibrations of C–H bonds in the –CH_2_ and –CH_3_ groups were observed at wavenumbers of 2934 cm^−1^ and 2860 cm^−1^, respectively. Additionally, it has been observed that there exists an absorption band at a wavenumber of 1693 cm^−1^, which can be attributed to the presence of C=O groups. Furthermore, another absorption band at a wavenumber of 1533 cm^−1^ has been identified as characteristic of urethane [35].

Based on the FTIR spectra analysis of four tannin-derived products obtained at ambient temperature, it is observed that all four reaction products exhibit a stretching vibration band at 1690 cm^−1^, which corresponds to the C=O double bond of amides [31,98,99]. Additionally, the presence of stretching bands at 3340 cm^−1^, corresponding to secondary amides, suggests the formation of NIPU. In contrast, the characteristic urethane band observed at 1537 cm^−1^ is more pronounced in the reaction products of the two Pine tannins compared to those of Quebracho tannins. The predominant constituents of Mimosa tannin are aromatic nuclei. with distinct peaks at 1600 cm^−1^, 1500 cm^−1^, and 1460 cm^−1^. This observation suggests that the presence of urethane connections is less relevant compared to the polyphenolic structures of flavonoids. Among the tannin sources, Quebracho tannins demonstrate higher equilibrated absorbances in the spectral bands located at 1690 cm^−1^, 1600 cm^−1^, 1537 cm^−1^, 1500 cm^−1^, and 1460 cm^−1^ [31,98,99,100,101]. 

The thermal stability of bio-based NIPU resins mostly depends on the quantity of aromatic moieties, due to their inherent resistance to degradation. The TGA investigation provides insights into the degradability and potential applications [78,79]. At a temperature of 210 °C, TGA was conducted, which resulted in the identification of a tannin curve exhibiting two distinct degradation phases: degradation of phenyl groups and degradation of carbonyl groups [3,29,32,33,36,91]. In contrast, the PU samples exhibited three distinct phases of degradation. The initial occurrence takes place at a temperature of 220 °C, specifically involving the breakdown of urethane bonds. The second phase of degradation is concomitantly linked with the deterioration of the tannin sample, transpiring within the identical temperature range spanning from 230 °C to 290 °C. The observed decrease in weight within this range of temperatures can be utilized to measure the tannin concentration in the bio-based NIPU. Specifically, as the concentration increased, the weight loss of the sample increased from 6.0% to 7.1%. In addition, it should be noted that the third stage of the degradation process takes place within a temperature range of 290 °C to 300 °C, during which the degradation of ester groups and carbonyl groups derived from tannins occurs [78,79]. The degradation process persisted until reaching a temperature of 430 °C. It is worth noting that the reaction products are more prone to producing NIPU oligomers when maintained at room temperature. At elevated temperatures, there is a tendency to undergo polymerization. The reaction might progress from 180 to 450 °C due to both the polymerization and degradation processes. At approximately 450 °C, the occurrence of thermo-oxidative degradation is highly probable. At a temperature of 700 °C, the remaining weight falls within the range of 22% to 35% and constitutes a component of the aromatic structures of flavonoid tannins [92].

The determination of the condensed tannin oligomer and its derivative condensation products was carried out by MALDI-TOF analysis [22,77,79]. The observed peaks at 433.2 Da, 439.2 Da, 451.8 Da, and 556.3 Da corresponded to the Fisetinidin, Robinetinidin, Catechin, and Delphinidin of tannin-based NIPU, as reported in published works [22,77,79]. The identification of oligomers produced from tannins can also be facilitated by the confirmation of urethane linkages through the application of FTIR and ^13^C NMR techniques. Condensed tannins have the ability to undergo chemical changes and reactions, resulting in the formation of oligomers, which are then utilized in the production of NIPU adhesives. The condensed tannin oligomers have the potential to undergo polymerization to form the NIPU adhesives. The extent of condensation of tannin oligomers during the reaction with DMC and HDMA is contingent upon the particular circumstances and reactions employed in the synthesis procedure. An increased condensation level of tannin reaction with DMC and HDMA often results in the elongation of polymer chains and enhanced interconnectivity, leading to the development of an NIPU material with greater rigidity and reduced flexibility [3,23]. Moreover, the polymerization reaction mechanism of tannins and hexamines is intricate. Crosslinkers refer to two distinct types of bonds that may be discerned, namely two variations of methylene-based reactive fragments derived from HMDA, which are CH_2_, NH, CH, and NH_2_. The MALDI-TOF spectrum exhibits distinct peaks at certain mass-to-charge ratios, including 578.3 Da, 582.4 Da, 591.2 Da, 604.3 Da, 611.3 Da, 615.4 Da, 619.5 Da, 623.2 Da, 631.3 Da, 638.4 Da, 768.7 Da, 796.5 Da, 878.6 Da, 906.6 Da, 954.5 Da, and 963.4 Da. These peaks provide evidence for the generation of reaction products resulting from the interaction between tannin, DMC, and HMDA [21,68,69].

The composition of the bio-PU created during the production process is determined using the ^13^C-NMR technique [70,72,73,75]. The ^13^C-NMR spectra of tannin-based NIPU exhibited relatively small tannin peaks. These peaks were discernible between the shoulders of tannins C5 and C7 at a chemical shift of 157 ppm. Additionally, broad peaks were observed in the range of 153–155 ppm, corresponding to the C9 flavonoid unit. Furthermore, the presence of a carbonyl group (C=O) indicated the presence of the urethane link. The presence of the aromatic ring in urethane is indicated by the minor peak observed at a chemical shift of 150 ppm. Consequently, it can be observed that the formation of the urethane linkage on the aromatic tannin ring has occurred. Moreover, the prominent and expansive peaks observed at 24 ppm can be attributed to the presence of the –CH_2_ group in HDMA. Additionally, the peak detected at 59 ppm can be ascribed to the carbohydrate group found in commercially accessible tannin extracts. Based on the aforementioned findings, it can be inferred that the synthesis of NIPU, utilizing condensed tannins, was successfully achieved through the reaction of tannin with DMC and HDMA during the manufacturing phase of the bio-based NIPU resin. In the context of NIPU synthesis using tannins, two possible reactions can occur with HDMA [70,72,73,75]:

(a) Interaction between HDMA and the hydroxyl group of the tannin. In this chemical process, two amine groups (–NH_2_) of HDMA undergo a direct interaction with the hydroxyl groups (–OH) present in the tannin molecule, creating amide linkages.
(b) The hydroxyl groups (–OH) of the tannin undergo chemical modification with DMC, creating carbonate groups (–C=O) on the tannin molecule. The reaction is commonly referred to as a “carbonate formation” reaction. Subsequently, the carbonated tannin can react with HDMA, resulting in the creation of urethane linkages.

It is expected that the NIPUs containing lignin and tannin as a base will undergo polymerization upon exposure to a temperature above 103 °C. Consequently, the molecular weight is within the range of 400 to 2500 Da. However, it is important to note that these compounds exhibit different characteristics. Notably, the spectra of the four tannin-based NIPUs exhibit strong peaks at 612 Da and 776 Da, which are not observed in the spectra of DMC and HMDA. The observed discrepancy of 164 Da can be elucidated by the conjugation of an HMDA moiety to a carbonated group by a urethane linkage, concomitant with the presence of a sodium cation. A study conducted on Quebracho and Mimosa tannin-derived spectra revealed the presence of a repeating sequence characterized by gaps between peaks ranging from 156 Da to 168 Da [22,77,79]. Tannin-based NIPUs probably undergo a curing process resulting in the formation of three-dimensional cross-linked complex polymers. 

The GPC chromatogram of Quebracho tannin at ambient temperature indicates that the tannin-based NIPU possesses a molecular weight exceeding 2000 Da, with a polydispersity index of 2.45. When comparing the GPC profiles of tannin-based NIPU heated at 103 °C, it is evident that the conditions under which the NIPU is prepared have a significant impact on improving its molecular weight [98]. The NIPU yielded a distribution of molecular weights, with approximately 50% of the species exhibiting molecular weights exceeding 3500 Da. In this case, the polydispersity index (PDI) value decreases to 1.94 in Quebracho tannin, suggesting that polymerization is more efficient at elevated temperatures. The PDI is decreased when smaller moieties or monomers react to form larger polymers with a narrower range of molecular weights. Typically, this occurs when the polymerization process is well-controlled and the resulting product is more homogenous. Similarly, the PDI for Mimosa and coastal pine are 1.75 and 1.79, respectively [22,77,79]. It is possible for the molecular weight of an NIPU to increase during both its preparation and hot pressing. Due to additional polymerization reactions, the molecular weight increases during the synthesis of NIPU. When NIPU is utilized as an adhesive in the hot pressing, the curing reaction of the NIPU may result in an increase in molecular weight [98].

## 4. Future Perspective

Despite the fact that NIPU research has made significant strides in recent years, the synthesis of NIPU continues to confront formidable obstacles, most notably the poor reactivity of the cyclic carbonate–amine reaction [102,103,104]. The comparison between linear polyesters/polyethers and polyamines in conventional PU and tannin- and lignin-based NIPU with respect to aromatic/heavily branched and crosslinked structures poses significant issues. NIPU can be synthesized through the utilization of bio-based polyols obtained from tannins, lignin, or other sustainable sources. The molecular structure between the urethane groups in NIPU has a higher degree of complexity compared to conventional PU. Tannin and lignin molecules frequently exhibit aromatic characteristics and possess several functional groups. The intricate nature of this phenomenon might give rise to the formation of interconnected or branched configurations, resulting in improved mechanical characteristics and high-performance NIPU adhesive.

Furthermore, a combination of high reaction temperatures and long reaction times is necessary to synthesize the NIPU adhesive, implying that curing the NIPU on the item would take a long period. The epoxide group can be used as a bridge to produce ambient-curable NIPU adhesives in order to accelerate the curing process. A novel NIPU-epoxy hybrid resin possesses the flexibility of conventional polyurethane resins as well as the physical–chemical properties of conventional epoxy–amine resins [105]. The synthesis consists of two steps: first, the preparation of amine-terminated NIPU, followed by chain extension with a small amount of epoxy resin. Because the epoxy–amine reaction is substantially more reactive than the cyclic carbonate–amine reaction, the addition of the epoxide group reduces the reaction time for synthesizing NIPU, and NIPU–epoxy hybrid resins also exhibit exceptional performance. However, the NIPU–epoxy hybrid resins were still organic solvent-based, and no aqueous hybrid resins are currently available.

Due to the demand for high-performance bio-based NIPU adhesives, it is anticipated that bio-resources such as adipic acid and 1,6-hexanediol will be available for polyol synthesis [106,107,108]. In the case of aromatic monomers, 2,5-furandicarboxylic acid and vanillic acid are the compounds with the greatest potential for use in the synthesis of polyester polyols and NIPU adhesives [109]. 2,5-bis-(hydroxymethyl)furan (BHMF) and 2,5-diformylfuran (DFF) are two additional bio-based polyols that are integral to the production of NIPUs. BHMF and DFF can be used as a starting material to synthesize a bio-polyol through reactions with a diol (e.g., ethylene glycol, propylene glycol) or a polyol (e.g., glycerol) to form a DFF-based polyol. The resulting polyol can be reacted with DMC and HDMA to produce NIPU. Both of the previously mentioned monomers are derived from 5-hydroxymethylfurfural, which is produced by dehydrating C6 carbohydrates [110,111]. The substantial literature on aliphatic and aromatic bio-based polyols presents the realistic potential for using entirely bio-based polyols on an industrial scale in the near future. 

## 5. Conclusions

Lignin and tannin have the potential to be used as an alternative source for polyols in the production of NIPUs as wood adhesives. The polycondensation route for the production of NIPUs involves the reactions between di-or polycarbamate and diols, di-or polycarbonates and amino alcohols or diamines, poly chloroformate and polyamines, and poly carbamoyl chloride and polyol. The carbonation of lignin and tannin with dimethyl carbonate (DMC), followed by a reaction with hexamethylene diamine (HMDA), led to the formation of lignin- and tannin-based NIPU adhesives. The end result is a polymeric substance with urethane links produced during the polymerization process.

Lignin- and tannin-based NIPU adhesives can be utilized as wood adhesives that have various advantages over traditional adhesives such as PU adhesives typically used in the fabrication of wood-based panels, particularly plywood, laminated veneer lumber, glue laminated lumber, and cross-laminated timber. NIPU-based wood adhesives can undergo further reactions during the press cycle in the hot press used to make wood-based panels. These processes can cause an increase in the molecular weight and crosslinking of the adhesive, which is required for strong and long-lasting connections between wood particles or veneers. The following reactions can occur during the application of NIPU adhesives in the preparation of wood composites:

- Urethane Formation: Urethane linkages are produced in NIPU adhesives by the reaction of the polyol’s hydroxyl groups and the diamine’s amino groups. Residual hydroxyl groups in the NIPU adhesive may react further with amino groups or other functional groups present in the adhesive under heat and pressure in the hot press, resulting in an increase in molecular weight and potentially increasing the adhesive’s strength.
- Crosslinking: The development of chemical links between distinct polymer chains is referred to as crosslinking. The NIPU adhesive can undergo crosslinking reactions during the hot press cycle, either through the reaction of remaining reactive urethane groups or through secondary reactions involving additional functional groups present in the adhesive formulation, such as epoxy. Crosslinking helps to increase mechanical qualities including toughness and resistance to water and heat.

## Figures and Tables

**Table 1:** Comparison of NIPU adhesives with the conventional PU [12,38,43,46,57].

Properties	NIPU Adhesives	PU Adhesives
Physical	Lower water resistance and poor dimensional stability.	High water resistance.
Mechanical	High ratio of hardness to softness yields acceptable mechanical characteristics.	Superior mechanical properties.
Bonding	Low resistance to delamination, reduced tear resistance, low cohesion and adhesion strength.	High resistance to delamination, as well as strong adhesive and cohesion strength.
Chemical	Poor chemical resistance.	Greater chemical resistance.
Thermal	The thermal stability is improved due to the existence of aromatic bio-polyphenolic compounds.	Higher thermal stability, due to the isocyanate.
Renewability	Derived from renewable biomass.	Derived from non-renewable petroleum sources.
Toxicity	Less toxicity, due to being isocyanate free.	Carcinogenic, due to isocyanate.

**Table 2:** Typical functional groups of lignin- and tannin-based NIPU resins using FTIR spectroscopy.

Type of NIPU Resins	Wavenumber (cm^−1^)	Functional Groups	References
Lignin-based NIPU	3400–3350	N–H stretching	[39,91,92,93,94,95,96,97]
	2950–2900	C–H stretching (CH_3_ and CH_2_)	
	2860–2840	C–H stretching (OCH_3_)	
	1720–1600	C=O of urethane	
	1600–1595	C=O of aromatic lignin	
	1515–1500	C–C aromatic ring	
	1240–1220	C–N of amine	
	1115	Aromatic C–H (syringol)	
	1085–1030	C–O–H aliphatic and C–O–C ether	
	1030	C-O in syringyl and guaiacyl	
Tannin-based NIPU	3500–3300	O–H stretching vibration	[3,32,33,34,35,98]
	3340–3200	N–H stretching	
	2970–2850	C–H stretching aliphatic groups	
	1630–1590	C=O of urethane	
	1260–1240	C–N of amine	
	1090	C–O–C of aliphatic	

## Data Availability

The data presented in this study are available on request from the corresponding author.
